# Deciphering the unexplored *Leptospira* diversity from soils uncovers genomic evolution to virulence

**DOI:** 10.1099/mgen.0.000144

**Published:** 2018-01-03

**Authors:** Roman Thibeaux, Gregorio Iraola, Ignacio Ferrés, Emilie Bierque, Dominique Girault, Marie-Estelle Soupé-Gilbert, Mathieu Picardeau, Cyrille Goarant

**Affiliations:** ^1^​ Institut Pasteur in New Caledonia, Institut Pasteur International Network, Leptospirosis Research and Expertise Unit, Noumea, New Caledonia; ^2^​ Institut Pasteur Montevideo, Bioinformatics Unit, Uruguay; ^3^​ Institut Pasteur, Unité de Biologie des Spirochètes, 28 rue du docteur Roux, 75724 Paris Cedex 15, France

**Keywords:** *Leptospira*, genomics, evolution, virulence, ecology

## Abstract

Despite recent advances in our understanding of the genomics of members of the genus *Leptospira*, little is known on how virulence has emerged in this heterogeneous bacterial genus as well as on the lifestyle of pathogenic members of the genus *Leptospira* outside animal hosts. Here, we isolated 12 novel species of the genus *Leptospira* from tropical soils, significantly increasing the number of known species to 35 and finding evidence of highly unexplored biodiversity in the genus. Extended comparative phylogenomics and pan-genome analyses at the genus level by incorporating 26 novel genomes, revealed that, the traditional leptospiral ‘pathogens’ cluster, as defined by their phylogenetic position, can be split in two groups with distinct virulence potential and accessory gene patterns. These genomic distinctions are strongly linked to the ability to cause or not severe infections in animal models and humans. Our results not only provide new insights into virulence evolution in the members of the genus *Leptospira*, but also lay the foundations for refining the classification of the pathogenic species.

## Data Summary

1. *Leptospira* isolates are described in Table S1 (available in the online version of this article) and locations of isolation are shown on Fig. S1.

2. Genomes have been deposited in GenBank; accession numbers are given in Table S2.

3. Overall genomic relatedness indices are presented in Tables S3 (ANI) and S4 (AAI).

4. The protein domain abundance matrix is shown in Table S6 (xls file).

**Impact Statement**

Water-associated exposures are the main risk factors for leptospirosis, a complex disease with a multitude of infecting serovars, a broad reservoir host range, non-specific clinical manifestations and difficult diagnosis. To assess the diversity of environmental members of the genus *Leptospira*, we isolated and sequenced members of the genus *Leptospira* from hot spots of leptospirosis. General analysis of these genomes provided unprecedented insight into the diversity of the genus *Leptospira*. We described a total of 12 novel species, including species belonging to the cluster of potentially infectious leptospires. Surprisingly, novel species from the pathogenic cluster failed to produce an infection in animal models. A detailed analysis of accessory genomes revealed clear differences within this pathogen cluster between virulent species and others failing to cause infection. This sheds new light into the evolution and acquisition of virulence in this highly heterogeneous genus.

## Introduction

Pathogenic species of the genus *Leptospira* cause leptospirosis, an emerging zoonosis worldwide with high prevalence in tropical low-income countries. Leptospirosis affects 1 million and kills 60 000 people annually, but remains poorly documented and often underestimated [1]. The burden of leptospirosis and its economic cost are significant and similar to that of other important neglected tropical diseases, including schistosomiasis, leishmaniasis and lymphatic filariasis [2]. Pathogenic leptospires are maintained in the renal tubules of asymptomatic reservoir animals, frequently rodents, and are excreted through the urine, contaminating the environment, where they can survive for months. Environment-mediated contamination is considered to be the major source of transmission to humans. Other animals, including livestock and companion animals, can also get infected and develop leptospirosis.

The genus *Leptospira* (phylum *Spirochetes*) is highly heterogeneous and genetically distinct from other bacteria, being currently divided into 22 species and more than 300 serovars. Phylogenetic analysis, initially based on the 16S rRNA gene but later on whole-genome sequences, showed that the genus is separated into three monophyletic clusters named ‘saprophytes’, ‘intermediates’ and ‘pathogens’. The ‘saprophytes’ are environmental species which are rapidly cleared in animal models, and are non-pathogenic to humans and other animals. The ‘intermediates’ have been recently described in both humans and animals, but infection of the classical animal models for acute leptospirosis with these species cannot reproduce the disease. Life-threatening species like *Leptospira interrogans*, which is the dominant pathogenic species worldwide, are classified within the ‘pathogens’ cluster and can infect every mammal. Conversely, *Leptospira kmetyi* belongs to the ‘pathogens’ cluster but has been isolated only from soil and never recovered from animals [3], calling the ecological coherence of the current classification into question.

The molecular bases of leptospiral pathogenicity, virulence and persistence remain at the onset of understanding, mainly because pathogenic species are fastidious and not prone to genetic manipulations, hampering the experimental discovery and validation of virulence determinants [4]. Alternatively, comparative genomics has uncovered key aspects of genomic adaptations to virulence [5, 6] but relevant questions still remain to be answered, fundamentally about the mechanisms that led the leptospiral ancestor to evolve from a saprophytic lifestyle into mammal-adapted pathogens. Consequently, a systematic evaluation of the relationship between genomic traits' evolution and virulence potential requires to be established [7].

In this work we reveal a significant amount of unexplored taxonomic diversity within the genus *Leptospira* by isolating 12 novel species from soils in areas of endemic leptospirosis. Using comparative phylogenetics, pan-genome analyses and *in vivo* models of infection we demonstrate that the ‘pathogens’ cluster is heterogeneous, being composed of both virulent and low-virulence strains with remarkable genomic distinctions. Our results provide new insights into virulence evolution in the genus *Leptospira* and indicate that the current classification of leptospiral species should be revised.

## Methods

### Ethics statement, patient contact and authorization for interview

Institut Pasteur in New Caledonia has been the country reference and only laboratory for the biological diagnosis of human leptospirosis from 1980 to 2016. The patients were identified by a positive diagnostic quantitative PCR and notified to the New Caledonian Health Authority, which also investigates cases through interviews. Oral consent was requested by the Health Authority to meet with the patient, visit and collect environmental samples in the suspected infection sites. The detailed procedure has been described previously [8].

### Study sites

Six sites were chosen based on the good acceptance of the project by the patients and custom chiefdom [Koné, Touho (two sites), Ponerihouen (two sites) and Yaté]. All sites were within Melanesian tribal areas and three (Koné and two sites in Touho) were also included in a previous study [8]. These sites are indicated on Fig. S1 together with the 30 year average temperatures (minima and maxima) and rainfall of the closest meteorological stations (retrieved from the Météo France free online public database).

### Collection and processing of environmental samples on site

*Leptospira* collection permits were obtained from the North (# 60912-2002-2017/JJC) and South (Arrêté 1689-2017/ARR/DENV) Provinces of New Caledonia. Environmental investigations were started a few weeks after the presumed human infection dates and after recovery of the patients, between March and June 2016. The soils selected to attempt culture of members of the genus *Leptospira* and isolation were chosen following discussions on site with the patients, based on environmental exposure of the patient on the day of probable contamination. The samples mostly included river soils, but also moist soils at a distance from any waterway if suggested by patient interviews (muddy walking tracks, agricultural soils). Most samples from the study sites were collected less than 20 meters one from one another; 27 soil samples were used to isolate members of the genus *Leptospira*. Samples were collected and processed on site as follows: approximately 5 g topsoil was collected from riverbanks (from 10 cm below to 1 m above water level), walking track or culture fields from a core sample (3 cm large by 5–7 cm height). Each soil sample was placed into a 15 ml sterile Falcon tube within 2 h of collection and vigorously shaken with 5–10 ml sterile water. The soil particles were allowed to settle for 5–15 min and 2 ml of supernatant were filtered through a sterile 0.45 µm filter into a tube filled with 2.5 ml of 2× EMJH medium. Alternatively, the process was repeated the next day at the laboratory, leaving more time for particles to settle, then culturing without filtration. Finally, we added 500 µl of 10× concentrated STAFF, a combination of selective agents for isolation of members of the genus *Leptospira* made of sulfamethoxazole, trimethoprim, amphotericin B, fosfomycin, and 5-fluorouracil [9]. Culture tubes from the field were transported within 12 h at ambient temperature to the laboratory, where they were put in an incubator at 30 °C. Alternatively, they were directly placed in the incubators when prepared in the laboratory.

### Leptospira isolation

Cultures were checked daily by dark-field microscopy for the growth of spirochetes. When contaminants were observed, the cultures tubes were subcultured with STAFF after filtration through a 0.45 µm membrane filter. When spirochetes were observed, a 50 µl and a 200 µl volume of the culture at various dilutions was plated onto EMJH agar and incubated at 30 °C until individual subsurface colonies were visible. Most of individual colonies started to appear after 3 days of incubation, and at day 10 all plates were positive with 10 to 100 colonies of members of the genus *Leptospira*. One to five characteristic subsurface individual colonies were collected from each plate for confirmation of a morphology typical of members of the genus *Leptospira* by dark field microscopy before clonal subculture in liquid EMJH (Table S1).

### Whole-genome sequencing

Genomic DNA was prepared by collection of cells by centrifugation from an exponential-phase culture and extraction with a MagNA Pure 96 Instrument (Roche). Next-generation sequencing was performed by the Mutualized Platform for Microbiology (P2M) at Institut Pasteur, using the Nextera XT DNA Library Preparation kit (Illumina), the NextSeq 500 sequencing system (Illumina) and the CLC Genomics Workbench 9 software (Qiagen) for analysis. The quality of the initial assemblies was improved with SPAdes [10] and a post-assembly improvement pipeline [11], the resulting draft genomes were automatically annotated with Prokka [12]. Draft genomes were submitted to Genbank, accession numbers are available in Table S2.

### Taxonogenomics, pan-genome and phylogenetic analyses

A comprehensive set of draft and closed genomes that represents the currently described leptospiral species was retrieved from the PATRIC database [13]. Most of these genomes have been previously used to study the genomic evolution of the genus *Leptospira* [5]. The final dataset was composed of available genomes (*n*=22, because whole genome sequences for *Leptospira idonii* were not publicly available, but including the reference genome of *Leptospira venezuelensis* sp. nov. currently under description by members of our group [14]) and those sequenced in this study (*n*=26).

To determine the relationship of each sequenced genome to previously described or novel leptospiral species, we calculated two Overall Genetic Relatedness Indices (OGRIs): the Average Nucleotide Identity (ANI) and the Average Amino acid Identity (AAI). Both indices were automatically calculated using two-way blast + blastn and blastp [15] comparisons as previously implemented [16], using the Taxxo R package (https://github.com/giraola/taxxo).

To build a standard phylogeny the 16S rRNA gene sequences were extracted from whole genomes (the genome of *Leptonema illini* DSM 21528^T^ was included as an outgroup) using blast + blastn against the 16S ribosomal RNA sequence database at the NCBI. Sequences were aligned with MAFFT [17] and phylogenetic reconstruction was performed with FastTree v.2.1 [18] using the GTR substitution model and 1000 replicates to calculate bootstrap values.

To build a genome-wide high-resolution phylogeny of the whole genus *Leptospira* and using the *Leptonema illini* DSM 21528^T^ genome as the outgroup (*n*=49), a set of highly conserved core genes (present in at least 95 % of the genomes) was identified by comparing each genome against the eggNOG v3.0 database [19] specifically customized for the phylum *Spirochaetes* (spiNOG) using HMMER v3.1b2 [20]. A set of 671 genes were identified, concatenated and aligned with MAFFT [17] (total alignment length was 778 190 bp). Phylogenetic reconstruction was performed as described above. Pairwise patristic distances were calculated from the resulting tree using the APE package [21].

Comparative pan-genome analyses were performed over the set of genomes belonging to the ‘intermediates’ (*n*=15) and ‘pathogens’ (*n*=17) clusters. The pan-genome was reconstructed using an in-house pipeline (available at https://github.com/iferres/pewit). Briefly, for every genome, each annotated gene was scanned against the Pfam database [22] using HMMER3 v3.1b2 hmmsearch [20] and its domain architecture was recorded (presence and order). A primary set of orthologous clusters was generated by grouping genes sharing exactly the same domain architecture. Then, remaining genes without hits against the Pfam database were compared with each other at protein level using HMMER3 v3.1b2 phmmer and clustered using the MCL algorithm [23]. These coarse clusters were then split using a tree-prunning algorithm which allows discrimination between orthologous and paralogous genes. Functional category assignments to each orthologous cluster were performed with blast + blastp against the Clusters of Ortholog Groups (COGs) database [24]. The Jaccard distance over accessory gene patterns was calculated with the package ade4 [25]. Cluster-defining accessory genes were identified with K-pax2 [26] by running its Bayesian clustering method over the pan-genome matrix with default parameters. Paralogous genes were defined as those orthologous clusters with more than one gene copy in at least one genome and the Bray–Curtis distance was calculated with the package Vegan [27]. Protein domains were extracted by comparing each genome annotation against the Pfam database [23] using HMMER3 v3.1b2 hmmsearch [20]. A domain abundance matrix was created by recording the number of occurrences of each domain in each genome and this was used to perform a Discriminant Analysis of Principal Components (DAPC) as implemented in package adegenet [28]. To identify genes contributing highly to the observed clustering we used the PCA loadings and adjusted them to a normal distribution. Then those genes with loadings departing more than two standard deviations (sd) from the mean were selected. Bray–Curtis distances from the abundance patterns of selected genes were calculated as explained above. Tests of proportions and Mann–Witney U were calculated in R [29].

### Virulence of novel species

To evaluate if isolates of the novel pathogenic and intermediate species were virulent, 7–8-week-old golden Syrian hamsters (males and females) and 8-week-old Oncins-France 1 (OF-1, outbred) mice (males and females) whose progenitors originate from Charles River Laboratories, were infected by intraperitoneal injection of 2×10^8^ leptospires in pure culture. Similar infections were performed with hamsters and mice, which were similarly infected with *Leptospira interrogans* serovar Manilae strain L495 (2×10^6^ per animal) and *Leptospira borgpetersenii* serogroup Ballum strain B3-13S (2×10^8^ per animal). Hamsters were euthanized by carbon dioxide inhalation 4.5 days after infection and the blood collected from heart puncture was cultured in EMJH. The urine of mice was collected 7–8 days after infection, DNA was extracted and analyzed by a real-time PCR targeting the conserved regions of the 16S rRNA gene *rrs* [30]. After two weeks, mice were euthanized by carbon dioxide inhalation. One kidney was collected and its DNA extracted and analyzed using the same PCR. All experiments were replicated twice on different days and using independent bacterial cultures. Animal experiments were conducted according to the guidelines of the Animal Care and Use Committees of the Institut Pasteur of Paris and of New Caledonia, and followed European Recommendation 2007/526/EC. Protocols and experiments were approved by the Animal Care and Use Committees of the Institut Pasteur in New Caledonia.

## Results

### Culture isolation, identification and phylogenetic position of novel species

Using a previously described [9] combination of selective agents that facilitates the isolation of leptospires from complex environmental samples, we isolated 26 strains of members of the genus *Leptospira* from tropical soils from six sites in New Caledonia, where the disease is endemic (Fig. S1 and Table S1).

**Fig. 1.**
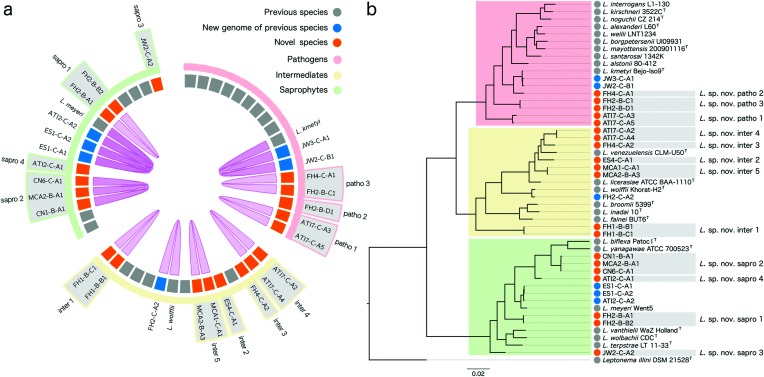
Phylogenetic position of the novel species. (a) Circos diagram showing the relationships between leptospiral genomes based on overall genomic relatedness indices (OGRIs). The inner violet ribbons connect pairs of genomes if they share >95 % average nucleotide identity (ANI) and average amino acid identity (AAI). Blocks represent each genome coloured as explained below. The outer highlights show the leptospiral clades. (b) Maximum-likelihood phylogeny for the genus *Leptospira* based on the core genome alignment. The tree is rooted with *Leptonema illini* DSM 21528^T^. The three classic leptospiral clades historically associated with differential pathogenicity are highlighted in red (‘pathogens’), yellow (‘intermediates’) and green (‘saprophytes’). Coloured circles at species labels indicate a public genome from a previously described species (grey), a genome sequenced in this study assigned to a previously described species (blue) or a genome sequenced in this study from a novel species (orange).

Whole-genome sequences of all 26 isolates were determined (genome statistics are presented in Table S2). To assign the novel species to the traditional leptospiral phylogenetic clusters we built a full-length 16S rRNA gene phylogeny (Fig. S2). By comparing the identity of full-length 16S rRNA gene sequences, we could assign a few isolates to previously described leptospiral species (100 % nucleotide identity). However, the remaining isolates had unique sequences, suggesting the presence of unknown species. We calculated both the average nucleotide identity (ANI) and the average amino acid identity (AAI) of the 26 isolates against each other and to the 22 previously described species of the genus *Leptospira* (see Methods and Tables S3 and S4). Fig. 1a shows the relationship between genomes according to the standard ANI and/or AAI threshold >95±1 %. This analysis confirmed the presence of 12 novel species of the genus *Leptospira*, thus extending by 55 % the number of species within this genus.

As 16S rRNA gene sequence conservation prevents precise separation of some well-defined species of the genus *Leptospira* [5], we then built a high-resolution phylogenetic tree based on the concatenated coding sequences of 671 leptospiral core genes (also occurring in *Leptonema illini*) (see Methods). This phylogeny not only reproduced the typical topology with the three main clusters designated as pathogens, intermediates and saprophytes but also confirmed the position of the 12 novel species as separate branches (Fig. 1b). Three of them were classified with the pathogens (later designated sp. nov. patho 1–3), five novel species were identified as intermediates (later designated sp. nov. inter 1–5) and four novel species were assigned to the saprophytes (later designated sp. nov. sapro 1–4). Interestingly, the three novel species assigned to the pathogens presented a basal position with respect to the previously identified species within this group and were closer to the tree root (Fig. S3).

### Accessory gene patterns recapitulate virulence potential

**Fig. 2.**
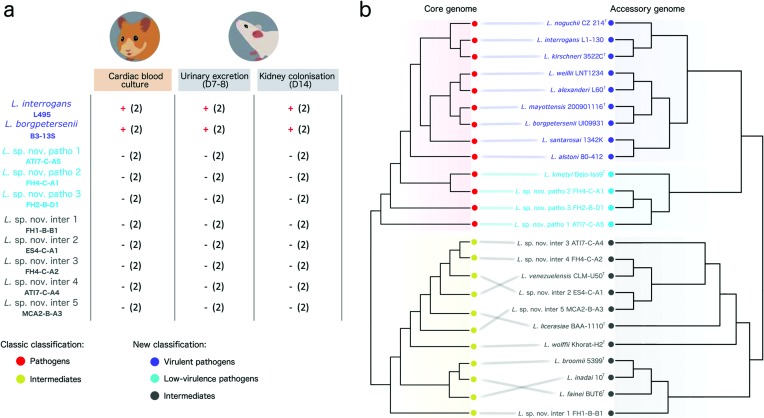
Virulence in animal models and accessory genome topology. (a) Virulence of novel species in experimental challenge infections (*n*=2 for each strain and animal model). Only the pathogenic strains *Leptospira interrogans* L495 and *Leptospira borgpeterseni* B3-13S were recovered from hamster cardiac blood or evidenced from mouse urine and kidney. (b) Tanglegram comparing the topology of the core genome phylogeny (left) and the topology obtained by clustering the genomes using Jaccard distance calculated over the accessory gene patterns (right). On the left, genomes are coloured according to the classic phylogenetic classification (only pathogens and intermediates are shown here). On the right, genomes are coloured according to the new classification based on accessory gene patterns.

The description of novel species assigned to both pathogens and intermediates prompted us to evaluate their relative virulence using animal models. Infection with virulent strains is associated with systemic infection with bacteremia and usually with severe acute disease in susceptible animals such as hamsters [31], and with an asymptomatic infection leading to renal colonization and urinary shedding in mice and rats [32]. Fig. 2a shows the infection profiles of one representative isolate per novel species identified as a member of the pathogens and intermediates, in comparison with the virulent references *Leptospira interrogans* strain L495 and *Leptospira borgpetersenii* strain B3-13S. The hamsters infected with these virulent strains showed signs of acute infection 3–4 days after infection (decreased activity, anorexia, ruffled fur and jaundice visible at the oral mucosa and skin levels) and renal colonization was evidenced in mice one and two weeks after infection. In contrast, hamsters infected with the novel species displayed no alteration in behavior, aspect or appetite and no culture was obtained from their blood and no leptospiral DNA was detected in the urine or the kidney of mice infected with the same strains. These results indicate the inability of these novel species to establish acute infection or renal colonization in these animal models. This is in marked contrast to the behavior of virulent pathogens like *Leptospira interrogans* and *Leptospira borgpetersenii*, suggesting the hereinafter denomination of these novel species as ‘low-virulence pathogens’.

These results led us to conduct a detailed comparative analysis of the accessory genomes of virulent pathogens, low-virulence pathogens and intermediates. We first noted that after adding the genomes from novel species belonging to these groups the pan-genome remained open (Fig. S4), revealing the divergent and highly diverse attributes of the members of the genus *Leptospira.* Then, a comparison of accessory gene patterns using the Jaccard distance showed a clear separation of intermediates from virulent and low-virulence pathogens (Fig. 2b). More interestingly, the accessory gene patterns were informative enough to discriminate two clusters that correlate with the subdivision of pathogens into virulent pathogens and low-virulence pathogens, in agreement with the virulence experiments. It is worth mentioning that *Leptospira kmetyi* belongs to the accessory genome cluster containing the low-virulence pathogens, which is also coherent with the unknown virulence potential of this species whose isolation has been only reported from soils. Hence, this analysis revealed clear genomic distinctions in the accessory genome of virulent and low-virulence pathogens that are not evident from the core genome phylogeny, which shows that low-virulence pathogens are a paraphyletic group (Fig. 2b).

### Genomic features associated with leptospiral virulence

**Fig. 3.**
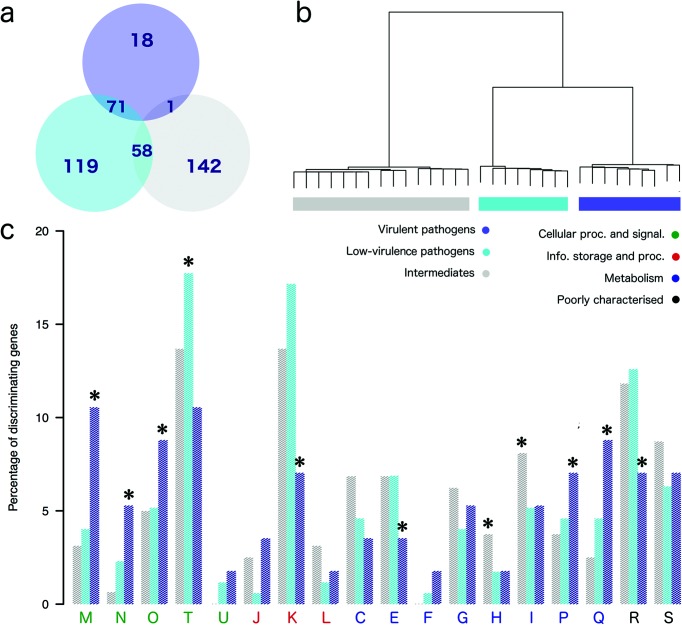
Functional analysis of discriminating accessory genes. (a) Venn diagram showing the Bayesian identification of cluster-defining accessory genes from the pan-genome. (b) Clustering analysis based on Jaccard distances calculated from the presence/absence vectors of cluster-defining genes. (c) Barplots showing the percentage of cluster-defining genes assigned to each COG functional category in each cluster. Statistical significance (*P*<0.001, test of proportions) is indicated with asterisks.

To provide a functional overview of the evolutionary adaptations associated with leptospiral virulence, we identified the genes that are associated with virulent or low-virulence pathogens. First, we used a Bayesian probabilistic framework [26] to detect those accessory genes with high discriminatory power for the three virulence groups (virulent pathogens, low-virulence pathogens and intermediates). Fig. 3a shows the number of discriminatory genes for each group (*n*=409), representing approximately 1.5 % of the accessory genes occurring in intermediates, low-virulence and virulent pathogens. Among these, 18 genes were found to distinguish virulent pathogens from both low-virulence pathogens and from intermediates (Table S5). Fig. 3b shows that using the presence/absence patterns of this small subset of genes completely recapitulates the three virulence groups, showing that very specific accessory genes can explain the evolution of virulence in the genus *Leptospira*. To gain insight into the biological functions related to this discrimination, we assigned COG [24] annotations to detect any functional enrichment. Fig. 3c shows that many functional categories are differentially represented in the accessory gene subsets defining each virulence group. Virulent pathogens are mainly distinguished from others by a significantly higher number of genes related to cell wall/membrane biogenesis (M), cell motility and chemotaxis (N), both known or suspected to be involved in virulence [33, 34], post-translational modification (O), also suspected to be involved in virulence [5, 35], as well as a lower number of genes related to amino acid metabolism and transport (E) and transcription (K). These differences reflect functional distinctions that are specific to virulent pathogens in comparison with both low-virulence pathogens and intermediates.

**Fig. 4.**
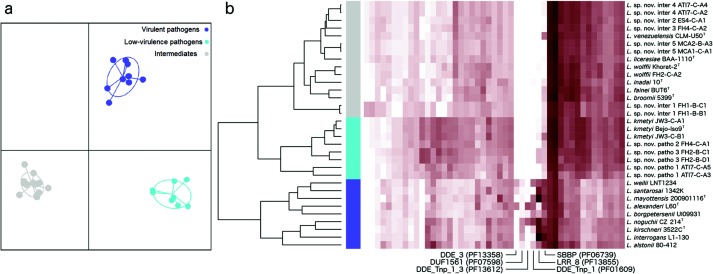
Protein domains analysis. (a) Scatterplot showing the first and second discriminant functions obtained from the Discriminant Analysis of Principal Components (DAPC), performed with protein domain abundances extracted from the coding sequences of each genome. Groups are coloured according to the new classification: intermediates (grey), low-virulence pathogens (cyan) and virulent pathogens (purple). (b) Heatmap showing the relationships between genomes obtained by calculating the Bray–Curtis distances from abundance patterns of a subset of highly discriminating domains obtained from the DAPC analysis. Redness indicates increasing domain copy number.

To have a more complete description of group-specific molecular functions associated with virulence, and considering the observed bias in COG annotations where a substantial proportion (44 %) of genes is not assigned to any known function, we analyzed the abundance patterns of protein domains by comparing each genome against the Pfam database [22]. Fig. 4a shows a Discriminant Analysis of Principal Components (DAPC) [28] that completely discriminates the three virulence groups using protein domain patterns. Furthermore, when considering only those domains that are highly informative for generating the observed clustering (see Methods), we were able to reproduce the three virulence groups using a different clustering analysis based on the Bray–Curtis distance (Fig. 4b). Interestingly, we noticed a group of six domains whose abundance was high in virulent pathogens while almost null in low-virulence pathogens and intermediates. Most of these domains belong to repeated elements such as mobile elements (DDE endonuclease superfamily) and proteins of paralogous families (Beta-propeller repeat- and leucine-rich repeat-containing proteins). This indicates that virulent pathogens can be distinguished by increased repeat sequence elements in comparison with the low-virulence pathogens and intermediates, suggesting a functional link to virulence. Other Pfam domains allowing this discrimination are presented in Table S6.

**Fig. 5.**
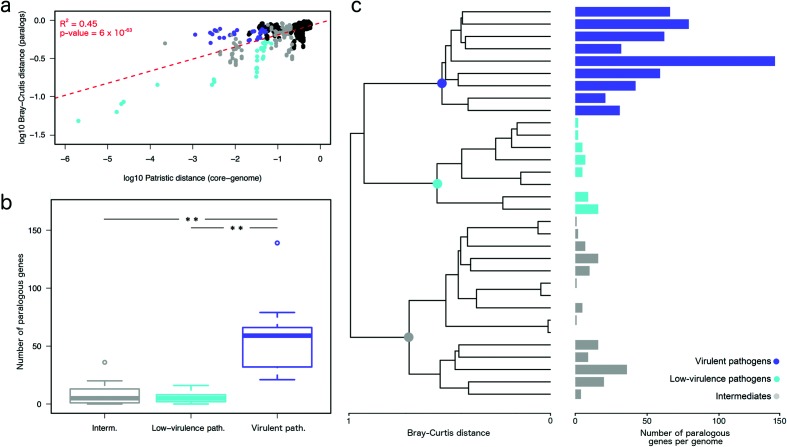
Analysis of paralogous genes. (a) Linear regression showing the correlation between patristic distances calculated from the core genome phylogeny and Bray–Curtis distances calculated from the abundance patterns of paralogous genes. Dots are coloured according to virulence clusters when both genomes in the pair belong to the same cluster, black dots represent pairs of genomes belonging to different groups. (b) Boxplots showing the distribution of paralogous genes in the three virulence clusters. Asterisks indicate *P*<0.001 (Mann–Witney U test). (c) Clustering analysis using the Bray–Curtis distances calculated from the abundance patterns of paralogous genes. Horizontal bars indicate the number of paralogous genes per genome and are coloured according to virulence clusters.

Given the importance of repeat sequence elements in ecological adaptation of organisms by shaping their genomes [36], an analysis focused on the abundance of paralogous genes in the accessory genomes was performed. First, we evidenced that patristic distances obtained from the core genome phylogeny were highly correlated with Bray–Curtis distances calculated from the abundance of paralogous genes (Fig. 5a), indicating that phylogenetically closer species share more similar paralogy patterns. Also, when observing just the abundance distributions of paralogous genes in each virulence group we detected a significantly higher incidence of paralogy in virulent pathogens in comparison with low-virulence pathogens and intermediates (*P*<0.001, Mann–Witney U test) (Fig. 5b). The same trend was observed in Fig. 5c, where Bray–Curtis distances were used to perform a cluster analysis that reconstructed the three virulence groups. Additionally, a significant and positive correlation was found between the number of transposase domains and the number of paralogous genes encoded in each genome (Fig. S5). In summary, these results indicate that paralogy has played an important role in the emergence of virulent pathogens.

## Discussion

Pathogenic species of the genus *Leptospira* are a unique group of highly fastidious bacteria, difficult to isolate in pure cultures. In this study, 12 novel species were successfully isolated from a relatively small number of soil samples from New Caledonia, highlighting a greatly unexplored biodiversity in the genus that is probably only the tip of the iceberg, and the number of recognized species may explode in a near future. Indeed, the presence of putatively novel uncultured species of the genus *Leptospira* has been detected from unrelated sources such as bats [37–41] and Amazonian soils [42]. The impressive diversity found in our study indicates that soils may not only be considered as a secondary passive reservoir of leptospirosis, but also the birthplace of the genus *Leptospira* as previously suggested [6].

From a medical point of view, the description of novel species of intermediates and pathogens may have implications for public health. However, in our infection experiments and despite high infectious doses, none of these novel species could induce signs or symptoms of infection in the hamster model, and isolates could not be recovered from hamster blood. Similarly, these isolates could not be detected in mouse urine or kidney, suggesting their inability either to infect mice or to colonize kidney tubules, also calling into question the need for a mammal reservoir in their biology. Moreover, only *Leptospira interrogans* and *Leptospira borgpetersenii* have been detected in clinical cases in New Caledonia through an active surveillance system [43, 44]. Together, these results indicate that the novel species have no or very limited virulence potential to mammals.

From an evolutionary perspective, genomes of these low-virulence species present an ancestral phylogenetic position with respect to the virulent pathogens, supporting the current hypothesis for explaining the emergence of leptospiral pathogens from free-living ancestral species inhabiting soils. This also indicates that virulence has evolved independently in pathogens and intermediates, as evidenced by different accessory gene and domain patterns in virulent pathogens and intermediates. More importantly, our results support the need to refine the classification of pathogens, which today are assumed to be an ecologically coherent group by sharing a higher virulence potential in comparison with intermediates. Despite the authors of some previous studies having proposed that virulence may be variable among different species classed as pathogens [5, 6, 45], our more comprehensive taxonomic coverage combined with infection experiments and accessory genome analyses demonstrated the presence of two groups of species of the genus *Leptospira* within the pathogens, correlated with clearly distinctive virulence potentials.

Taken together, our results indicate that virulent pathogens have adapted their genomes from a soil free-living to a mammal-associated virulent lifestyle mainly by expanding particular groups of protein families through gene duplication. These genomic distinctions should be used to establish more adequate criteria for the classification of pathogenic leptospires and to focus future work on the dissection of the molecular mechanisms and biological role of these genes.

## Data bibliography

1. Bateman A, Coin L, Durbin R, Finn RD, Hollich V *et al*. The Pfamprotein families database. *Nucleic Acids Res* 2004;32:138D–141.
2. Fouts DE, Matthias MA, Adhikarla H, Adler B, Amorim-Santos L *et al*. What makes a bacterial species pathogenic?: comparative genomic analysis of the genus *Leptospira*. *PLoS Negl Trop Dis* 2016;10:e0004403.
3. Galperin MY, Makarova KS, Wolf YI, Koonin EV. Expanded microbial genome coverage and improved protein family annotation in the COG database. *Nucleic Acids Res* 2015;43:D261–D269.
4. Puche R, Ferres I, Caraballo L, Rangel Y, Picardeau M *et al*. *Leptospira venezuelensis* sp. nov., a new member of the intermediates group isolated from rodents, cattle and humans. *IJSEM*. doi:10.1099/ijsem.0.002528 [Epub ahead of print].

## Supplementary Data

2871 Supplementary File 1

## Supplementary Data

686 Supplementary File 2
