# Supplementary material for: Deciphering the unexplored Leptospira diversity from soils uncovers genomic evolution to virulence
Source: Microb Genom. 2018 Jan 3;4(1):e000144. doi: 10.1099/mgen.0.000144 (PMC5857368; doi:10.1099/mgen.0.000144)
Supplement: Supplementary File 1 [file mgen-4-144-s001.pdf]

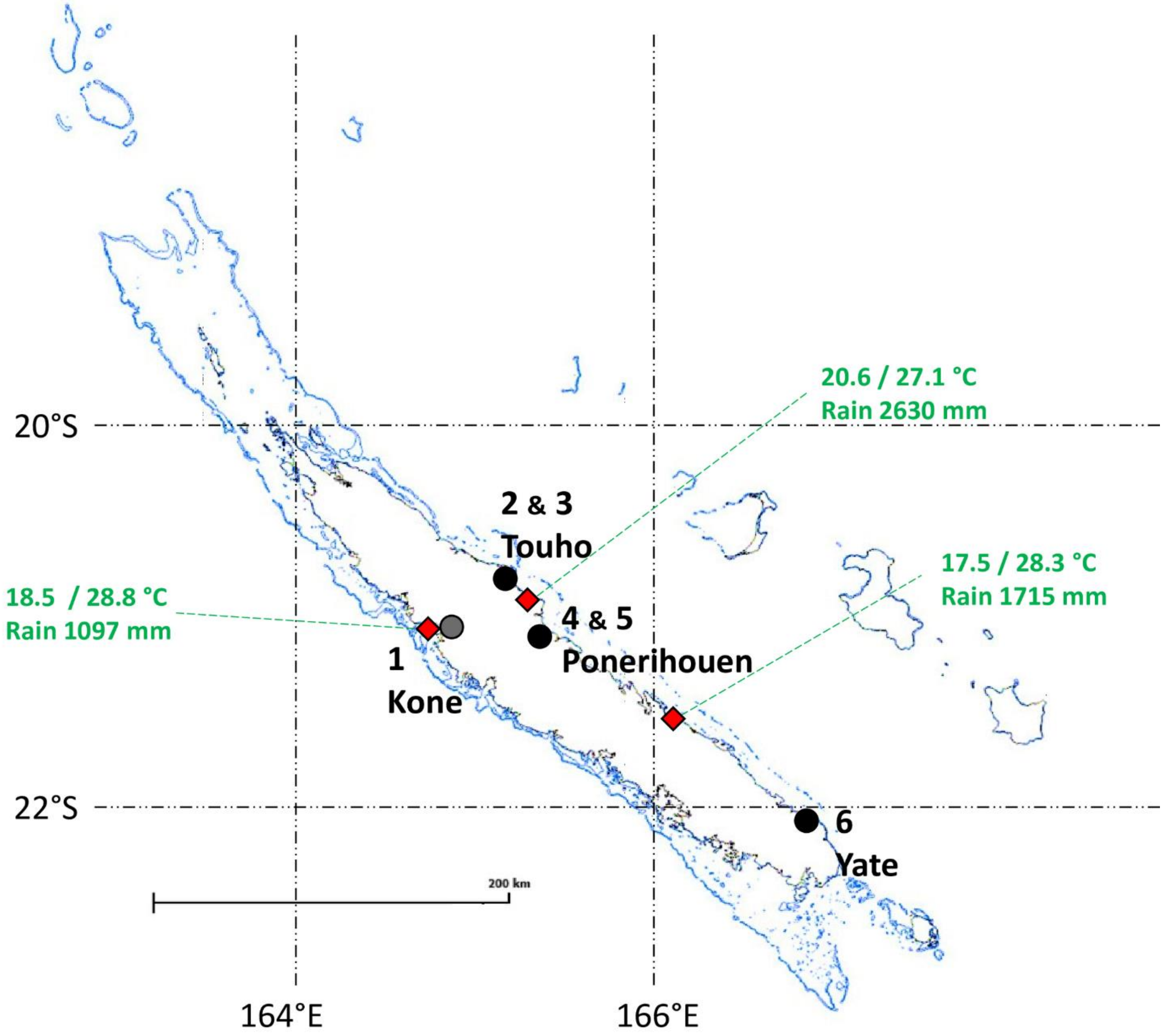

Supplementary Figure 1: Location of the soil collection areas in New Caledonia (circles) and meteorological data from closest station (red diamonds). The shoreline is black, blue lines are coral reefs. Temperature (mini / maxi) and rainfall data are the normal data calculated as the 30-year averaged data retrieved from the Meteo France public website. Black circles correspond to hotspots of human leptospirosis. The grey circle points to an area of lower incidence.

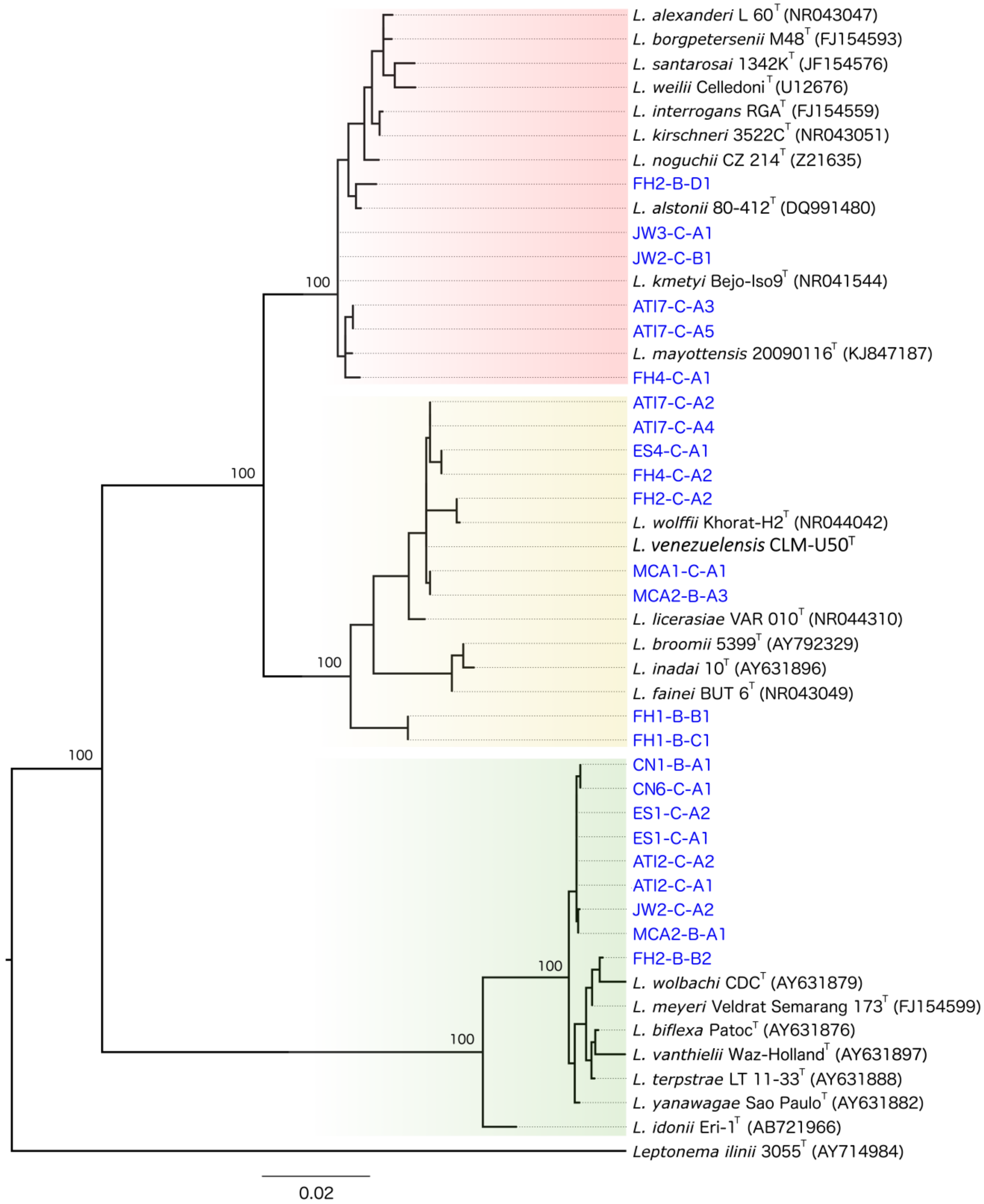

Supplementary Figure 2: Phylogenetic analysis using the 16S rRNA gene. Phylogenetic tree built with 16S reference sequences from described leptospiral species (black labels) and from the strains described in this work (blue labels). Color shades highlight the three *Leptospira* clades: pathogens (red), intermediates (yellow) and saprophytes (green). Bootstrap values are indicated for relevant nodes. For reference sequences, accession numbers are within brackets. The tree was rooted with *Leptonema ilinii*.

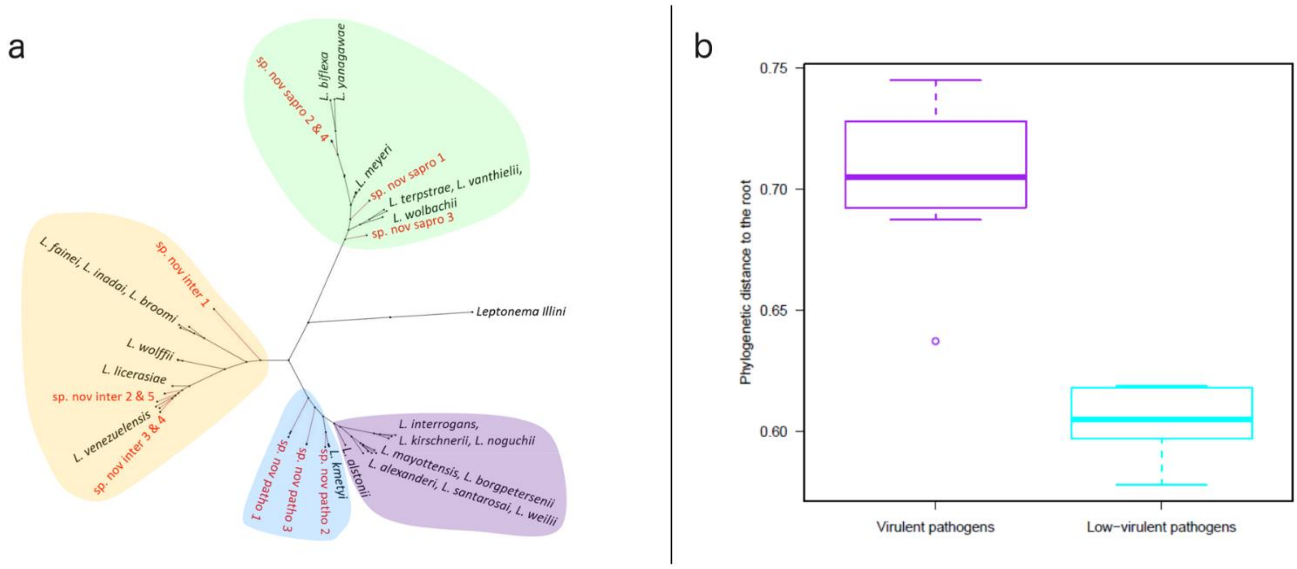

Supplementary Figure 3: (A) A radial view of the core genome topology showing novel species in all three *Leptospira* clusters. (B) Statistical evidence of the basal position of low-virulent species within the cluster "pathogens".

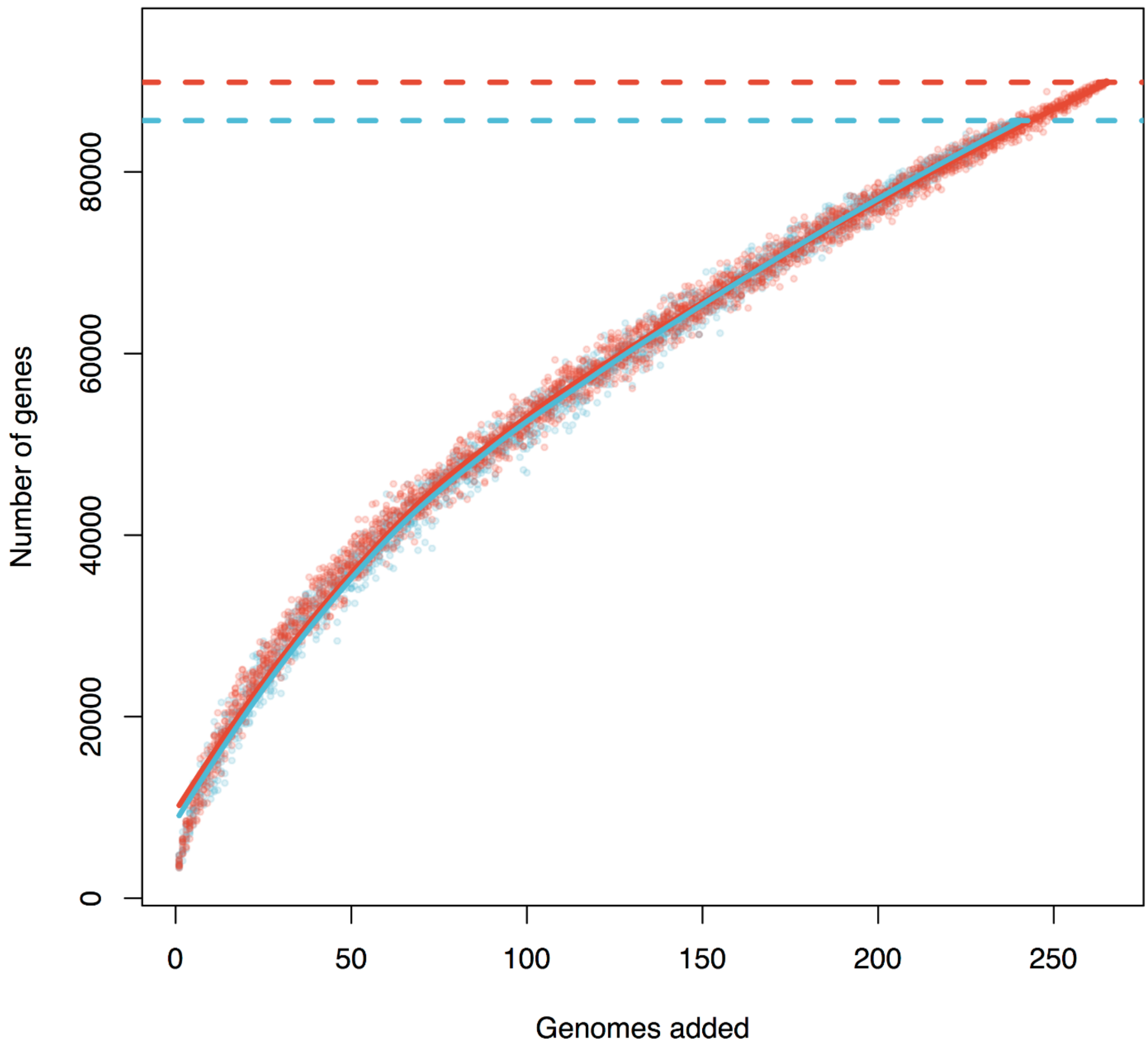

Supplementary Figure 4: Pan-genome size estimation. Gene abundance cumulative curves showing the estimated pan-genome size for a set of 240 public leptospiral genomes (blue) and for these genomes plus those sequenced in the present study (red). For the public dataset we estimated the pan-genome in 85,645 orthologous groups while when adding the genomes from novel species it increased to 89,868 orthologous groups. Dots show estimations for 10 random samples and solid lines represent the mean.

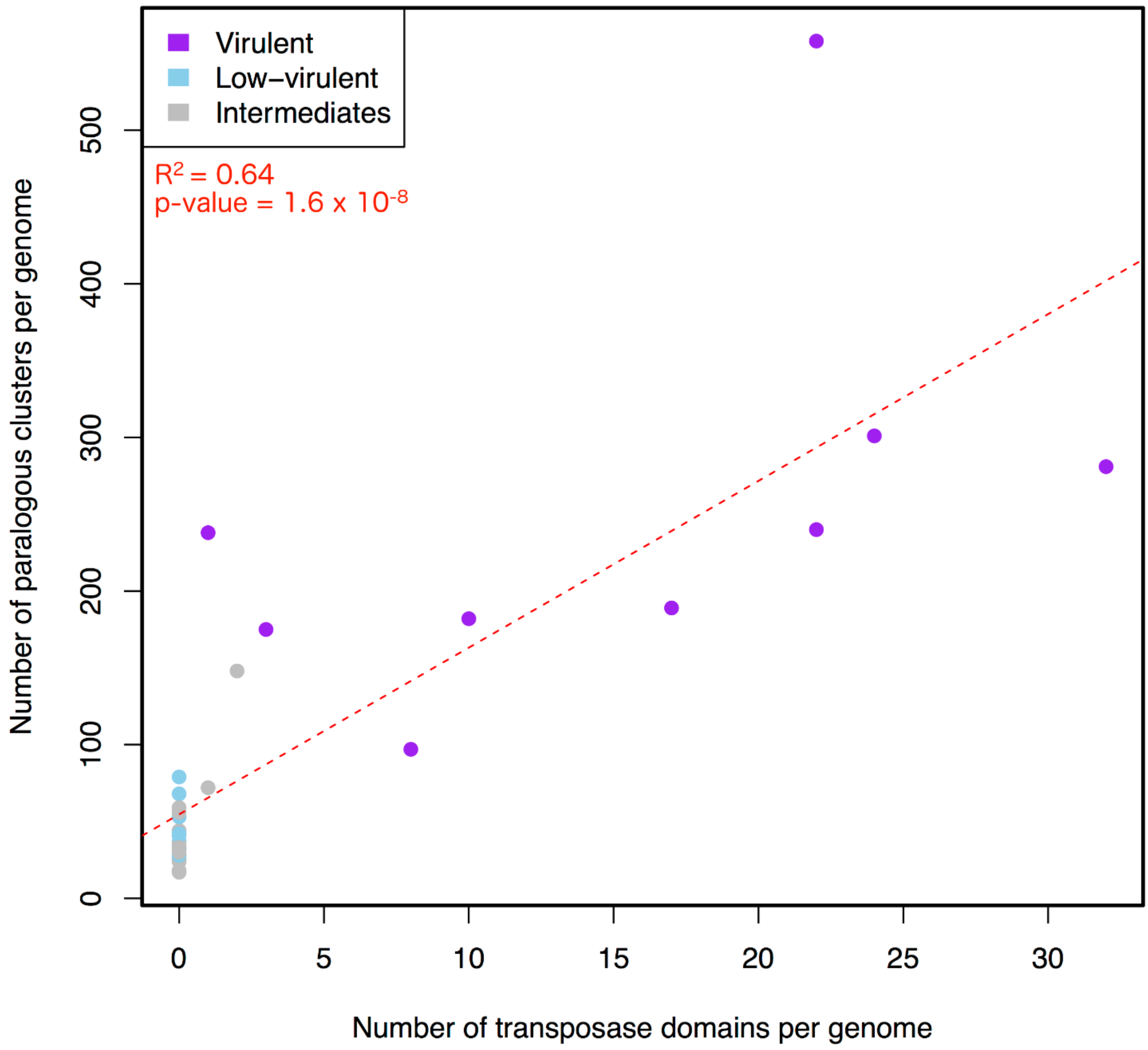

Supplementary Figure 5: Comparative abundance of paralogous clusters and transposase domains in the genomes of *Leptospira* from the intermediate cluster and two sub-clusters of pathogens.

Supplementary Table 1: Summary of information on samples, isolation process and identification of isolates.

Location	Sampling date	GPS coordinates	Sample name	Sample information	pH (water / KCl)	EMJH liquid + STAFF (time after sampling)	EMJH (agar plate)	Isolated colony	*Lepstospira* species identified
Ponerihouen North	30/05/2016	21°06S 165°18E	MCA1	wet soil on walking track	6.95 / 6.53	at the lab (next day)	#C	A1	***sp. nov. inter5***
			MCA2	wet soil on walking track	6.21 / 5.31	on site (<2 hours)	#B	A1	***sp. nov. sapro2***
								A3	***sp. nov. inter5***
Ponerihouen South	30/05/2016	21°06S 165°18E	CN1	wet soil close to small stream	n.d.	on site (<2 hours)	#B	A1	***sp. nov. sapro2***
			CN6	wet soil close to pig pen	n.d.	at the lab (next day)	#C	A1	***sp. nov. sapro2***
Touho North	31/05/2016	20°48S 165°09E	FH1	river bank (submerged)	n.d.	on site (<2 hours)	#B	B1	***sp. nov. inter1***
								C1	***sp. nov. inter1***
			FH2	river bank (submerged)	6.415 / 4.13	at the lab (next day)	#C	A2	***L. wolfii***
				river bank (submerged)		on site (<2 hours)	#B	A1	***sp. nov. sapro1***
								B2	***sp. nov. sapro1***
								C1	***sp. nov. patho3***
								D1	***sp. nov. patho3***
			FH4	river bank (above water level)	6.41 / 5.38	at the lab (next day)	#C	A1	***sp. nov. patho2***
								A2	***sp. nov. inter3***
Touho South	31/05/2016	20°49S 165°15E	JW2	river bank (submerged)	5.35 /4.46	at the lab (next day)	#C	A2	***sp. nov. sapro3***
								B1	***L. kmetyi***
			JW3	river bank (submerged)	5.93 / 4.49	at the lab (next day)	#C	A1	***L. kmetyi***
Koné	13/06/2016	21°00S 164°52E	ES4	river bank (sandy, above water level)	7.01 / 6.35	at the lab (next day)	#C	A1	***sp. nov. inter2***
			ES1	river bank (submerged)	6.88 / 6.51	at the lab (next day)	#C	A1	***L. meyeri***
								A2	***L. meyeri***
Unia	23/06/2016	22°06S 166°56E	ATI2	wet cultivated soil	6.79 / 6.51	at the lab (next day)	#C	A1	***sp. nov. sapro 4***
								A2	***L. meyeri***
			ATI7	river bank (submerged)	n.d.	at the lab (next day)	#C	A2	***sp. nov. inter4***
								A3	***sp. nov. patho1***
								A4	***sp. nov. inter4***
								A5	***sp. nov. patho1***

Supplementary Table 2. Genome summary statistics for the novel strains sequenced in the present study.

Strain	GC (%)	Total length	N50	Number of contigs	Largest contig	Number of CDS	Accession number
ATI2-C-A1	37,93	4223300	54433	295	280795	3856	NPEK00000000
ATI2-C-A2	38,11	4200752	63932	185	192006	3929	NPEJ00000000
ATI7-C-A2	39,81	4191631	324807	28	1067840	3861	NPEI00000000
ATI7-C-A3	44,75	4085768	8416	712	46328	4174	NPEH00000000
ATI7-C-A4	39,8	4191261	236721	34	1177136	3845	NPEG00000000
ATI7-C-A5	47,8	4314057	14342	517	47379	4159	NPEF00000000
CN1-B-A1	37,5	4264788	32869	278	114830	4037	NPEE00000000
CN6-C-A1	37,56	3900982	718917	31	817829	3626	NPED00000000
ES1-C-A1	38,13	4127728	422838	37	1503392	3892	NPEC00000000
ES1-C-A2	38,13	4115339	422838	37	625886	3881	NPEB00000000
ES4-C-A1	40,17	4213808	452736	31	537703	3932	NPEA00000000
FH1-B-B1	42,36	3985918	332586	39	819540	3637	NPDZ00000000
FH1-B-C1	42,36	3984751	170089	54	711234	3646	NPDY00000000
FH2-B-A1	37,86	3946761	2088660	14	2088660	3692	NPDX00000000
FH2-B-B2	37,76	4074637	2124476	18	2124476	3870	NPDW00000000
FH2-B-C1	43,82	4388198	202560	50	470705	3646	NPDV00000000
FH2-B-D1	43,55	4826170	61157	158	175409	4401	NPDU00000000
FH2-C-A2	45,81	4235896	338989	26	1517457	3939	NPDT00000000
FH4-C-A1	43,96	4392226	347630	16	977769	4045	NPDS00000000
FH4-C-A2	39,11	4083319	551160	32	1069366	3779	NPDR00000000
JW2-C-A2	38,34	4113151	489086	28	704882	3885	NPDQ00000000
JW2-C-B1	44,44	4650714	83791	102	281772	4274	NPDP00000000
JW3-C-A1	45,18	4706662	326698	94	788239	4351	NPDO00000000
MCA1-C-A1	40,47	4048089	406654	25	649247	3735	NPDN00000000
MCA2-B-A1	37,61	3875926	772998	13	1625029	3632	NPDM00000000
MCA2-B-A3	40,47	4051781	492748	25	606397	3740	NPDL00000000

Supplementary Table 3. Average Nucleotide Identity (ANI) values for the novel genomes against each other and previously identified species.

	ATI2-C-A1	ATI2-C-A2	ATI7-C-A2	ATI7-C-A3	ATI7-C-A4	ATI7-C-A5	CN1-B-A1	CN6-C-A1	ES1-C-A1	ES1-C-A2	ES4-C-A1	FH1-B-B1	FH1-B-C1	FH2-B-A1	FH2-B-B2	FH2-B-C1	FH2-B-D1	FH2-C-A2	FH4-C-A1	FH4-C-A2	JW2-C-A2	JW2-C-B1	JW3-C-A1	MCA1-C-A1	MCA2-B-A1	MCA2-B-A3
**ATI2-C-A1**	100	96.5	78.2	79.2	78.5	78.5	92.8	92.9	95.8	95.8	77.3	78.7	79	85.5	85.6	78.8	77.2	79.1	77.8	77.5	83.7	78.6	77.9	77.7	92.9	77.9
**ATI2-C-A2**	96	100	79.5	81.6	79.6	80.7	82.1	82.1	99.5	99.5	77.2	79.2	80.5	86.8	86.8	79	78.7	79.9	79.2	78.3	84.5	78.9	79.5	77.7	82.1	77.9
**ATI7-C-A2**	78.2	79.5	100	78.4	99.8	77.8	78.1	78.9	79.9	80	87.9	77.8	78	79	79.4	78.3	78	79.6	78	89.7	78.2	78.2	78.2	88.5	77.7	88.5
**ATI7-C-A3**	79.2	81.6	78.4	100	78.2	99.7	80.1	81.5	81.3	80.7	78	78	78.2	82.8	84.1	81.5	81.4	77.7	81.8	78.5	80.9	82.2	82.2	79.7	77.3	78.7
**ATI7-C-A4**	78.5	79.6	99.8	78.2	100	77.8	78.3	79	80.3	80.4	87.9	77.9	78.1	79.2	79.5	78.4	78.1	79.6	78.1	89.7	78	78.3	78.3	88.5	78.1	88.5
**ATI7-C-A5**	78.5	80.7	77.8	99.7	77.8	100	79.6	80.9	81.3	80.6	77.9	77.6	77.8	82.3	83.5	81.4	81.3	77.4	81.7	78.3	79.6	82	82.1	79.2	77	78.8
**CN1-B-A1**	92.8	82.1	78.1	80.1	78.3	79.6	100	99.7	82.1	82.1	79.4	80.8	80.9	82.1	82.2	80.3	78.1	82	79.9	78.5	82	81.5	81.2	78.5	99.7	79.1
**CN6-C-A1**	92.9	82.1	78.9	81.5	79	80.9	99.7	100	82.1	82.1	79.6	81	81.1	82.1	82.1	80.5	77.9	82.9	79.7	79	82	81.3	80.5	78.3	99.8	78.8
**ES1-C-A1**	95.8	99.5	79.9	81.3	80.3	81.3	82.1	82.1	100	100	78.9	80	81	86.8	86.7	80.1	79.4	81.2	78.9	78.5	84.6	79.4	78.4	78.1	82	78
**ES1-C-A2**	95.8	99.5	80	80.7	80.4	80.6	82.1	82.1	100	100	78.7	79.4	80.6	86.7	86.7	81.3	80.7	81.8	79.3	78.7	84.6	79	78.5	78.5	82.1	78.4
**ES4-C-A1**	77.3	77.2	87.9	78	87.9	77.9	79.4	79.6	78.9	78.7	100	78.1	78.3	78.5	79.9	78	77.9	79.8	77.9	88.3	78.8	78.1	77.8	88	78	88
**FH1-B-B1**	78.7	79.2	77.8	78	77.9	77.6	80.8	81	80	79.4	78.1	100	99.8	80.8	81.3	78.1	77.8	78.2	77.8	77.9	79.1	78	78.3	77.9	80.3	77.8
**FH1-B-C1**	79	80.5	78	78.2	78.1	77.8	80.9	81.1	81	80.6	78.3	99.8	100	81.5	81.5	78.4	78	78.3	77.8	78.2	79.4	78	78.2	78	79.3	77.8
**FH2-B-A1**	85.5	86.8	79	82.8	79.2	82.3	82.1	82.1	86.8	86.7	78.5	80.8	81.5	100	100	80.9	80.5	80.8	80.1	79	84.4	80.9	79.9	79	82.1	78.7
**FH2-B-B2**	85.6	86.8	79.4	84.1	79.5	83.5	82.2	82.1	86.7	86.7	79.9	81.3	81.5	100	100	81.3	80.7	83.3	80.8	79.6	84.5	81.6	81.2	80.4	82.3	79.8
**FH2-B-C1**	78.8	79	78.3	81.5	78.4	81.4	80.3	80.5	80.1	81.3	78	78.1	78.4	80.9	81.3	100	99.9	78	82.5	78.2	79.8	82.7	82.7	77.8	79.6	78.1
**FH2-B-D1**	77.2	78.7	78	81.4	78.1	81.3	78.1	77.9	79.4	80.7	77.9	77.8	78	80.5	80.7	99.9	100	77.9	82.4	77.9	78.6	82.6	82.6	77.7	77.9	78
**FH2-C-A2**	79.1	79.9	79.6	77.7	79.6	77.4	82	82.9	81.2	81.8	79.8	78.2	78.3	80.8	83.3	78	77.9	100	77.3	79.4	79.2	77.4	77.5	79.6	79.1	79.6
**FH4-C-A1**	77.8	79.2	78	81.8	78.1	81.7	79.9	79.7	78.9	79.3	77.9	77.8	77.8	80.1	80.8	82.5	82.4	77.3	100	78.1	79.8	90.6	90.6	78	78.1	78.2
**FH4-C-A2**	77.5	78.3	89.7	78.5	89.7	78.3	78.5	79	78.5	78.7	88.3	77.9	78.2	79	79.6	78.2	77.9	79.4	78.1	100	79.7	78.4	78.2	89	77.8	89
**JW2-C-A2**	83.7	84.5	78.2	80.9	78	79.6	82	82	84.6	84.6	78.8	79.1	79.4	84.4	84.5	79.8	78.6	79.2	79.8	79.7	100	78.9	78.6	78.5	82	77.8
**JW2-C-B1**	78.6	78.9	78.2	82.2	78.3	82	81.5	81.3	79.4	79	78.1	78	78	80.9	81.6	82.7	82.6	77.4	90.6	78.4	78.9	100	99.1	78.3	80.7	78.1
**JW3-C-A1**	77.9	79.5	78.2	82.2	78.3	82.1	81.2	80.5	78.4	78.5	77.8	78.3	78.2	79.9	81.2	82.7	82.6	77.5	90.6	78.2	78.6	99.1	100	77.7	77.5	77.7
**MCA1-C-A1**	77.7	77.7	88.5	79.7	88.5	79.2	78.5	78.3	78.1	78.5	88	77.9	78	79	80.4	77.8	77.7	79.6	78	89	78.5	78.3	77.7	100	77.7	99.9
**MCA2-B-A1**	92.9	82.1	77.7	77.3	78.1	77	99.7	99.8	82	82.1	78	80.3	79.3	82.1	82.3	79.6	77.9	79.1	78.1	77.8	82	80.7	77.5	77.7	100	78.2
**MCA2-B-A3**	77.9	77.9	88.5	78.7	88.5	78.8	79.1	78.8	78	78.4	88	77.8	77.8	78.7	79.8	78.1	78	79.6	78.2	89	77.8	78.1	77.7	99.9	78.2	100
***L. alexanderi* L60**	78.9	80.8	77.6	81.2	77.6	81	80.3	80.3	81.6	81.1	77.5	78.7	79.1	80.4	82.8	81.6	81.5	77.4	83.1	77.4	81.8	83.2	83.2	77.9	79.5	77.7
***L. alstonii* 80-412**	81.5	82.4	78.8	81.9	79.2	81.7	82.7	83.4	81.9	82	78.8	79.4	79	82.7	84.6	82.4	82.3	77.8	84.2	79.8	82	84.3	84.3	79.4	80.9	78.6
***L. biflexa* Patoc**	83.3	82.3	78.8	82.8	79.1	82.2	84.2	84.3	82.3	82.3	80.1	81.6	82.5	82.2	82.2	80.5	80	79.6	80.6	79.3	82	81	80.3	79.8	84.2	79.8
***L. borgpetersenii* UI09931**	78	80.4	77.6	81.2	77.8	81	81.2	79.8	79.4	80.2	77.1	77.6	77.8	78.6	80	81.4	81.3	77.8	82.9	77	78.7	83	83	78.3	79.8	78.2
***L. broomii* 5399**	80.6	82.7	78.2	78.6	78.1	78.5	82.8	86.5	82.5	82.1	78.3	78	78.1	82.9	85.8	77.6	77.3	78.3	77.4	78.3	81	78.1	77.4	78.2	83.5	78.2
***L. fainei* BUT6**	83.3	85.4	78	78.1	78.1	77.8	82.1	82.6	84.1	85.9	78.2	77.7	77.8	84.4	86.4	78.1	77.8	78.6	77.2	77.8	80.8	78.2	77.5	78.3	80.4	78.3
***L. inadai* 10**	86.3	88.2	78.7	79.3	78.6	79.1	84.1	85.2	86.3	85.8	78.8	78.8	78.8	86	87.2	79	78.9	78.6	78.1	78.3	83.1	78.3	77.3	78.7	83.9	78.6
***L. interrogans* L1-130**	78.9	79.7	78.2	80.9	78.4	80.5	80.6	81	80.2	80.1	78.7	78.2	78.5	80.2	81.6	80.8	80.8	79.1	81.8	78.3	79.5	81.8	81.8	78.5	78.8	78.4
***L. kirschneri* 3522**	78.1	79.2	78	80.7	78.3	80.4	79.8	80	79.4	79.5	77.7	78.2	78.7	79.3	80.1	80.8	80.8	77.3	82	77.8	79.5	81.9	81.9	78.1	77.9	78.1
***L. kmetyi* Bejo-Iso9**	79.3	82	78.4	82	78.5	81.9	81.8	81.7	81.8	81.7	78.1	78.5	78.4	82.1	83.6	82.7	82.6	77.7	90.7	78.2	80.5	99.1	99.1	78.2	79.1	78.1
***L. licerasiae* ATCC BAA-1110**	76.8	77.4	87.6	78.5	87.6	78	77.8	78.1	78	77.6	87.2	78	78.2	78.5	79.7	77.9	78	79.8	78.1	87.8	79.6	78.5	78.1	88.4	77.8	88.4
***L. mayottensis* 200901116**	79.1	82	77.9	81.1	77.9	80.9	80.6	79.7	83.4	82.6	78	77.8	78	80	81.4	81.5	81.3	78	82.8	77.4	80.3	82.9	82.8	78	77.3	77.9
***L. meyeri* Went5**	93.5	96.6	79.8	82.5	80.1	81.1	82	82.1	96.5	96.6	79.1	80.8	81.9	86.7	86.7	80.6	78.7	81.3	80.4	80.4	84.5	80.9	80.3	80.1	82	79.8
***L. noguchii* CZ214**	77.6	78.4	77.3	80.6	77.5	80.4	79.9	79.7	78.5	78.4	77.5	77.5	77.5	79.4	80.2	80.6	80.6	77.8	81.9	77.1	78.8	81.8	81.8	77.3	78.8	77.4
***L. santarosai* 1342K**	82.7	85.1	79	81.7	79.3	81.4	84.6	84.5	84.8	86.1	78.6	79	79.1	84.3	85.5	81.8	81.7	77.8	83.2	78.8	83.8	83.4	83.3	79.5	83.5	79.1
***L. terpstrae* LT11-33**	83.5	84.2	81	85	80.7	84	82.2	82.1	84.2	84.2	81.7	81.6	82.5	84.4	84.4	82.6	80.2	87.3	82.5	81.1	84.2	84.2	81.3	80.8	82.1	80.8
***L. vanthielii* Waz-Holland**	83.6	84.3	80.6	83.2	80.3	84.1	82	82	84.3	84.3	81.1	82.6	83.3	84.4	84.4	82.5	81.1	82.2	82.6	80.6	84.5	81.8	80.1	81.2	82	81
***L. venezuelensis* CLM-U50**	77.5	78.5	88.3	78.9	88.3	78.3	78.7	78.8	78	78.2	87.2	78	78.2	78.1	79.2	78.5	78.4	79.7	78	88.8	78.4	78.3	77.9	88	77.5	88
***L. weilii* LNT1234**	80.1	83	78.5	81.3	79.1	81	82.4	82.3	82.2	84.1	78.5	79.7	79.8	81.2	83.7	81.8	81.7	78.2	83	78.8	82.1	83.2	83.2	79	80.5	78.5
***L. wolbachii* CDC**	83.6	84.3	80.4	83	80.5	82.7	82	82.1	84.3	84.3	81.5	83.4	85	84.3	84.4	82.3	81.1	80.9	81.3	80.4	84.4	82.1	81	80.7	82	80.4
***L. wolffii* Khorat-H2**	80.1	81.1	79.6	78.3	79.6	77.9	82.1	82.8	81.6	82.4	79.7	77.8	77.9	81.6	83.1	78.6	78.5	96.8	77.9	79.4	80.4	77.8	77.8	79.6	80	79.7
***L. yanagawae* ATCC 700523**	83.3	82.2	78.6	84.7	79.2	82.7	84.1	84.2	82.2	82.2	79.4	82.8	83.2	82.2	82.2	82.3	81.1	82.1	80.3	79.1	81.9	82.8	81.8	78.9	84.2	79.7

Supplementary Table 4. Average Amino acid Identity (AAI) values for the novel genomes against each other and previously identified species.

	ATI2-C-A1	ATI2-C-A2	ATI7-C-A2	ATI7-C-A3	ATI7-C-A4	ATI7-C-A5	CN1-B-A1	CN6-C-A1	ES1-C-A1	ES1-C-A2	ES4-C-A1	FH1-B-B1	FH1-B-C1	FH2-B-A1	FH2-B-B2	FH2-B-C1	FH2-B-D1	FH2-C-A2	FH4-C-A1	FH4-C-A2	JW2-C-A2	JW2-C-B1	JW3-C-A1	MCA1-C-A1	MCA2-B-A1	MCA2-B-A3
**ATI2-C-A1**	**100**	92,6	64,1	64,1	64	64,2	88,5	88,5	92,5	92,5	64,2	64,1	64	87,9	88	64,2	64,1	64	64,1	64,2	85,7	64,2	64,1	64,2	88,6	64,1
**ATI2-C-A2**	92,6	**100**	64,2	64,4	64,1	64,2	81,8	82	**98,5**	**98,5**	64,2	64,2	64,1	91,1	91	64,2	64,3	64,1	64	64,2	87,5	64,2	64	64,2	82	64,1
**ATI7-C-A2**	64,1	64,2	**100**	68,2	**98,1**	67,8	64	63,8	63,9	63,9	92,4	71,9	72	63,8	63,8	67,8	67,6	78,3	67,6	93,5	63,8	67,9	67,8	92,7	64	92,8
**ATI7-C-A3**	64,1	64,4	68,2	**100**	67,9	**97**	63,7	64	64,2	64	68	68	67,9	64,2	64,5	78,7	79	68,3	79,8	68,1	64,3	79,7	79,6	67,9	64,2	67,8
**ATI7-C-A4**	64	64,1	**98,1**	67,9	**100**	67,7	63,9	64	63,9	64	92,5	72	71,9	63,8	63,8	67,8	67,7	78,5	67,7	93,7	64	67,9	67,8	92,9	64	92,9
**ATI7-C-A5**	64,2	64,2	67,8	**97**	67,7	**100**	64,4	63,9	64	64	67,5	67,8	67,5	63,8	63,7	78,5	78,7	67,7	79,3	67,5	64,1	79,5	79,3	67,6	64,3	67,5
**CN1-B-A1**	88,5	81,8	64	63,7	63,9	64,4	**100**	**98,4**	82,2	82,1	64,2	64,3	64,3	82,3	82,2	64,2	64	64,2	64,2	64,2	82	64,1	64,1	64,3	**98,2**	64,3
**CN6-C-A1**	88,5	82	63,8	64	64	63,9	**98,4**	**100**	81,8	81,9	63,9	63,9	63,9	82,1	82,1	64	63,9	63,8	63,9	63,8	81,8	64,1	63,9	63,9	**99**	63,9
**ES1-C-A1**	92,5	**98,5**	63,9	64,2	63,9	64	82,2	81,8	**100**	**99,1**	64,1	64	64	91,2	91,1	64	64	63,9	63,8	63,9	87,7	63,8	63,8	64	81,9	63,9
**ES1-C-A2**	92,5	**98,5**	63,9	64	64	64	82,1	81,9	**99,1**	**100**	64,1	64,1	63,9	91,2	91,1	63,9	63,8	64	63,8	64	87,8	63,8	63,8	64,1	81,9	64
**ES4-C-A1**	64,2	64,2	92,4	68	92,5	67,5	64,2	63,9	64,1	64,1	**100**	72,1	72	63,8	63,9	67,8	67,6	78,6	67,8	92,6	64	67,9	67,8	92,4	64	92,5
**FH1-B-B1**	64,1	64,2	71,9	68	72	67,8	64,3	63,9	64	64,1	72,1	**100**	**97,8**	63,9	63,9	68,3	68,1	72,4	67,9	72	63,7	68	67,8	72	64	72
**FH1-B-C1**	64	64,1	72	67,9	71,9	67,5	64,3	63,9	64	63,9	72	**97,8**	**100**	63,9	63,9	68,2	68	72,4	68	72,1	63,6	68	67,8	72	64	72
**FH2-B-A1**	87,9	91,1	63,8	64,2	63,8	63,8	82,3	82,1	91,2	91,2	63,8	63,9	63,9	**100**	**99,1**	63,9	63,9	63,8	64	63,8	87,6	64	63,9	63,9	82,2	63,8
**FH2-B-B2**	88	91	63,8	64,5	63,8	63,7	82,2	82,1	91,1	91,1	63,9	63,9	63,9	**99,1**	**100**	64	63,9	63,8	63,9	63,8	87,5	64	63,9	63,9	82,3	63,9
**FH2-B-C1**	64,2	64,2	67,8	78,7	67,8	78,5	64,2	64	64	63,9	67,8	68,3	68,2	63,9	64	**100**	**97,8**	68,3	81,6	67,8	63,9	81,2	81,4	67,8	64,1	67,8
**FH2-B-D1**	64,1	64,3	67,6	79	67,7	78,7	64	63,9	64	63,8	67,6	68,1	68	63,9	63,9	**97,8**	**100**	67,9	81,5	67,4	63,8	81,3	81,4	67,5	64	67,5
**FH2-C-A2**	64	64,1	78,3	68,3	78,5	67,7	64,2	63,8	63,9	64	78,6	72,4	72,4	63,8	63,8	68,3	67,9	**100**	67,8	78,4	63,8	67,9	67,7	78,5	63,7	78,5
**FH4-C-A1**	64,1	64	67,6	79,8	67,7	79,3	64,2	63,9	63,8	63,8	67,8	67,9	68	64	63,9	81,6	81,5	67,8	**100**	67,4	63,8	92,1	92,3	67,5	64,1	67,5
**FH4-C-A2**	64,2	64,2	93,5	68,1	93,7	67,5	64,2	63,8	63,9	64	92,6	72	72,1	63,8	63,8	67,8	67,4	78,4	67,4	**100**	64	67,7	67,6	92,9	63,9	93
**JW2-C-A2**	85,7	87,5	63,8	64,3	64	64,1	82	81,8	87,7	87,8	64	63,7	63,6	87,6	87,5	63,9	63,8	63,8	63,8	64	**100**	63,9	63,9	64,1	81,8	63,9
**JW2-C-B1**	64,2	64,2	67,9	79,7	67,9	79,5	64,1	64,1	63,8	63,8	67,9	68	68	64	64	81,2	81,3	67,9	92,1	67,7	63,9	**100**	**97,3**	67,5	64,1	67,6
**JW3-C-A1**	64,1	64	67,8	79,6	67,8	79,3	64,1	63,9	63,8	63,8	67,8	67,8	67,8	63,9	63,9	81,4	81,4	67,7	92,3	67,6	63,9	**97,3**	**100**	67,7	64,1	67,6
**MCA1-C-A1**	64,2	64,2	92,7	67,9	92,9	67,6	64,3	63,9	64	64,1	92,4	72	72	63,9	63,9	67,8	67,5	78,5	67,5	92,9	64,1	67,5	67,7	**100**	63,8	**98,4**
**MCA2-B-A1**	88,6	82	64	64,2	64	64,3	**98,2**	**99**	81,9	81,9	64	64	64	82,2	82,3	64,1	64	63,7	64,1	63,9	81,8	64,1	64,1	63,8	**100**	63,8
**MCA2-B-A3**	64,1	64,1	92,8	67,8	92,9	67,5	64,3	63,9	63,9	64	92,5	72	72	63,8	63,9	67,8	67,5	78,5	67,5	93	63,9	67,6	67,6	98,4	63,8	**100**
***L. alexanderi* L60**	64,6	64,8	68,1	79,5	68,2	79,7	65,1	64,5	64,3	64,5	68,2	68,6	68,7	64,3	64,5	81,2	81,1	68,3	84,7	68,2	64,2	84,6	84,4	68,1	64,3	68
***L. alstonii* 80-412**	64,3	64,4	67,6	79,2	67,8	78,5	64,3	64	64,1	64	67,7	68,3	68,2	64	63,9	80,5	80,4	68	84,4	67,8	63,8	84,2	83,9	67,7	64,3	67,8
***L. biflexa* Patoc**	84,3	82,1	63,9	64,2	63,9	64,1	87,1	87,1	82	82	64,1	63,8	63,9	82	82,2	64	63,7	63,9	64	64	82	64,1	64	63,9	87	63,9
***L. borgpetersenii* UI09931**	64,5	64,5	68	80,1	68,1	80,2	64,5	64,1	64,1	64,1	68,2	68,4	68,2	64,2	64	81,9	81,6	68,4	84,6	67,9	64,3	84,8	84,6	68	64,1	68
***L. broomii* 5399**	63,8	63,7	73,8	67,8	73,8	67,8	63,8	63,4	63,6	63,5	74	73	73,1	63,8	63,7	68	67,8	74,4	67,7	73,8	63,8	67,8	67,8	73,6	63,6	73,5
***L. fainei* BUT6**	63,7	63,7	73,6	68,2	73,6	67,9	63,9	63,6	63,7	63,6	73,9	73	72,8	63,7	63,7	68	67,8	74,3	67,6	73,7	63,7	67,8	67,8	73,6	63,6	73,7
***L. inadai* 10**	59,9	60,2	60,3	60,5	60,3	59,9	60,2	60	59,9	60	60,1	60,1	60,2	59,9	59,9	59,7	59,7	59,9	60	60,1	59,6	59,9	60,2	60	59,9	60
***L. interrogans* L1-130**	63,8	63,7	73,8	68,2	73,9	67,8	63,9	63,5	63,6	63,6	74	72,9	72,9	63,8	63,8	69,3	69,4	74,3	67,8	73,8	63,8	67,9	67,7	73,7	63,6	73,7
***L. kirschneri* 3522**	64,2	64,3	67,8	78,8	67,8	78,6	64,2	64,1	64,2	64,2	67,9	68,2	68,2	64	64	79,7	79,6	68,1	82,8	67,7	64,1	82,8	82,7	67,9	64,2	67,9
***L. kmetyi* Bejo-Iso9**	64,2	64,6	68	79,2	68	78,7	64,3	63,9	64,2	64,1	68,1	68,2	68,4	64	64,1	80,2	80,2	68,3	83,2	67,7	64	83,3	83	67,9	63,9	68
***L. licerasiae* ATCC BAA-1110**	64,1	64,2	67,6	79,9	67,6	79,3	64	63,9	63,8	63,8	67,7	67,9	67,9	64	63,9	81,4	81,4	67,8	92,4	67,5	63,9	**97**	**96,7**	67,5	64,1	67,5
***L. mayottensis* 200901116**	64,1	64	92,2	68,1	92,3	67,7	64,3	64	63,7	63,8	91,8	72	72,1	63,8	63,8	67,7	67,4	78,5	67,7	92,3	63,8	67,5	67,5	93,2	64	93,2
***L. meyeri* Went5**	64,4	**97,1**	67,9	79,6	68	79,9	64,5	64	**97,2**	**97,1**	68	68,1	68,1	64	64,1	81,3	81,3	68	84,6	67,9	64	84,6	84,5	67,8	64,1	67,8
***L. noguchii* CZ214**	91,7	64,5	63,9	64	63,9	64	82,1	82	64,1	64,1	63,9	64	64	91,1	91	64	63,8	63,8	63,7	63,9	87,6	63,9	63,7	64	82	63,9
***L. santarosai* 1342K**	64,5	64,6	67,8	79,1	67,9	78,9	64,6	64,3	64,3	64,2	67,9	68,2	68,3	64,2	64,2	79,5	79,5	68,2	83	67,8	64	82,6	82,8	67,8	64,2	67,8
***L. terpstrae* LT11-33**	64,3	64,3	68	79,4	68,1	79	64,2	64	64	64,1	67,9	68,4	68,3	64,1	64,2	81,1	80,8	68,3	83,9	67,9	64,2	83,9	83,9	68	64,1	67,9
***L. vanthielii* Waz-Holland**	85,8	87,8	63,8	63,9	63,7	64	82,3	81,8	87,9	87,9	63,8	64	64	88,1	88	63,9	63,8	63,8	63,7	63,6	87,5	63,9	63,6	63,8	82	63,7
***L. venezuelensis* CLM-U50**	85,6	87,9	63,9	63,7	64,1	63,8	81,9	81,9	87,9	87,9	64	63,8	63,9	88	88	63,9	63,9	63,8	63,5	63,8	87,7	63,7	63,8	63,9	82	63,7
***L. weilii* LNT1234**	64,1	64	92,5	68,1	92,5	67,6	64,2	63,9	63,9	63,9	91,6	71,9	71,9	63,8	63,7	67,8	67,5	78,2	67,6	92,7	63,8	67,7	67,7	92,5	63,9	92,6
***L. wolbachii* CDC**	64,6	64,7	68,3	79,8	68,5	79,6	64,7	64	64,2	64,4	68,4	69,2	69,2	64,4	64,1	81,4	81,4	68,6	84,2	68,3	64	84,5	84,4	68,1	64,2	68,1
***L. wolffii* Khorat-H2**	85,9	87,9	63,8	64,3	63,8	63,8	82,1	81,8	88,1	88,2	64	64	64,1	88,2	88,2	63,9	63,9	63,8	63,7	63,9	87,9	63,9	64	64	82	63,9
***L. yanagawae* ATCC 700523**	64	63,9	78	67,8	78,2	68,4	64,2	63,7	63,8	63,8	78,4	72,4	72,3	63,7	63,6	68	67,8	**96,7**	67,6	78,3	63,9	67,7	67,8	78,6	63,7	78,5

Supplementary Table S5. List of 18 genes found to discriminate virulent pathogens from low-virulent pathogens and intermediates

**Orthologous group ID**	**Genbank GI number**	**Locus**<br>(*L. interrogans* Lai strain 56601 genome)	**Annotation**
**OG00224**	NP_713138	LA_2958	alpha/beta hydrolase
**OG01961**	NP_711893	LA_1712	signal peptidase I
**OG02015**	NP_710323	LA_0142	PilZ domain-containing protein
**OG02077**	NP_712822	LA_2641	ferrichrome-iron receptor
**OG02081**	NP_712423	LA_2242	TonB-dependent receptor
**OG02787**	NP_710509	LA_0328	Acyltransferase
**OG03125**	NP_711583	LA_1402	DUF1561 domain-containing protein
**OG03945**	NP_712621	LA_2440	cytoplasmic membrane protein
**OG03987**	NP_712686	LA_2505	Esterase/lipase
**OG04438**	NP_714014	LA_3834	hypothetical protein
**OG04439**	NP_710464	LA_0283	beta-propeller repeat protein
**OG04918**	NP_713420	LA_3240	hypothetical protein
**OG05354**	NP_713091	LA_2910	hypothetical protein
**OG05579**	NP_710673	LA_0492	Lipoprotein LipL36
**OG07609**	NP_712431	LA_2250	Endonuclease S1/P1
**OG08282**	NP_710321	LA_0140	tRNA (guanine-N(7)-)-methyltransferase
**OG09184**	NP_712201	LA_2020	hypothetical protein
**OG09220**	NP_710851	LA_0670	hypothetical protein
